# Supplementary figures and images for: Population birth outcomes in 2020 and experiences of expectant mothers during the COVID-19 pandemic: A ‘born in Wales’ mixed methods study using routine data
Source: PLoS One. 2022 May 24;17(5):e0267176. doi: 10.1371/journal.pone.0267176 (PMC9129046; doi:10.1371/journal.pone.0267176)

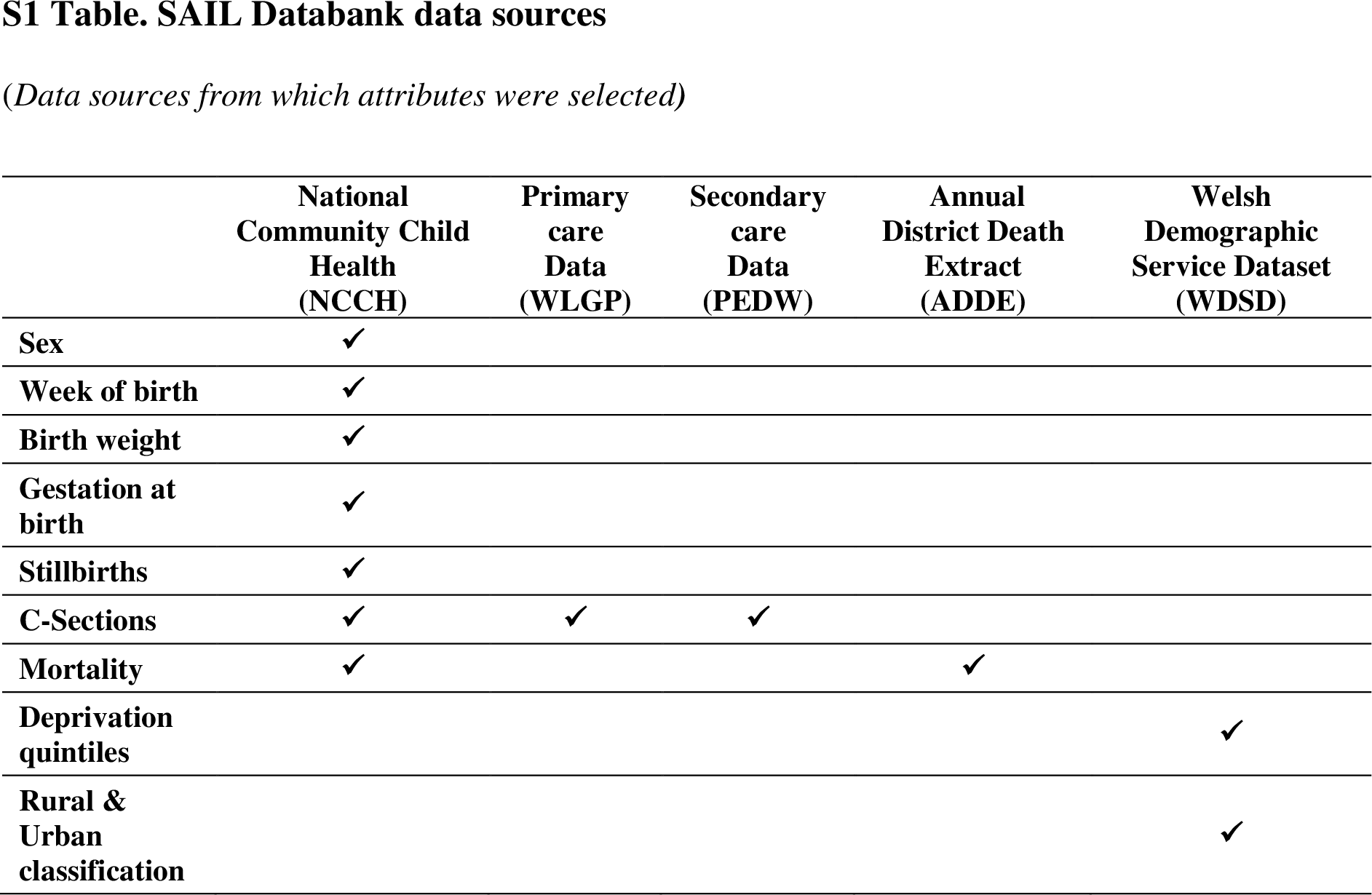

Supplement: S1 Table — (TIF) [file pone.0267176.s001.tif]

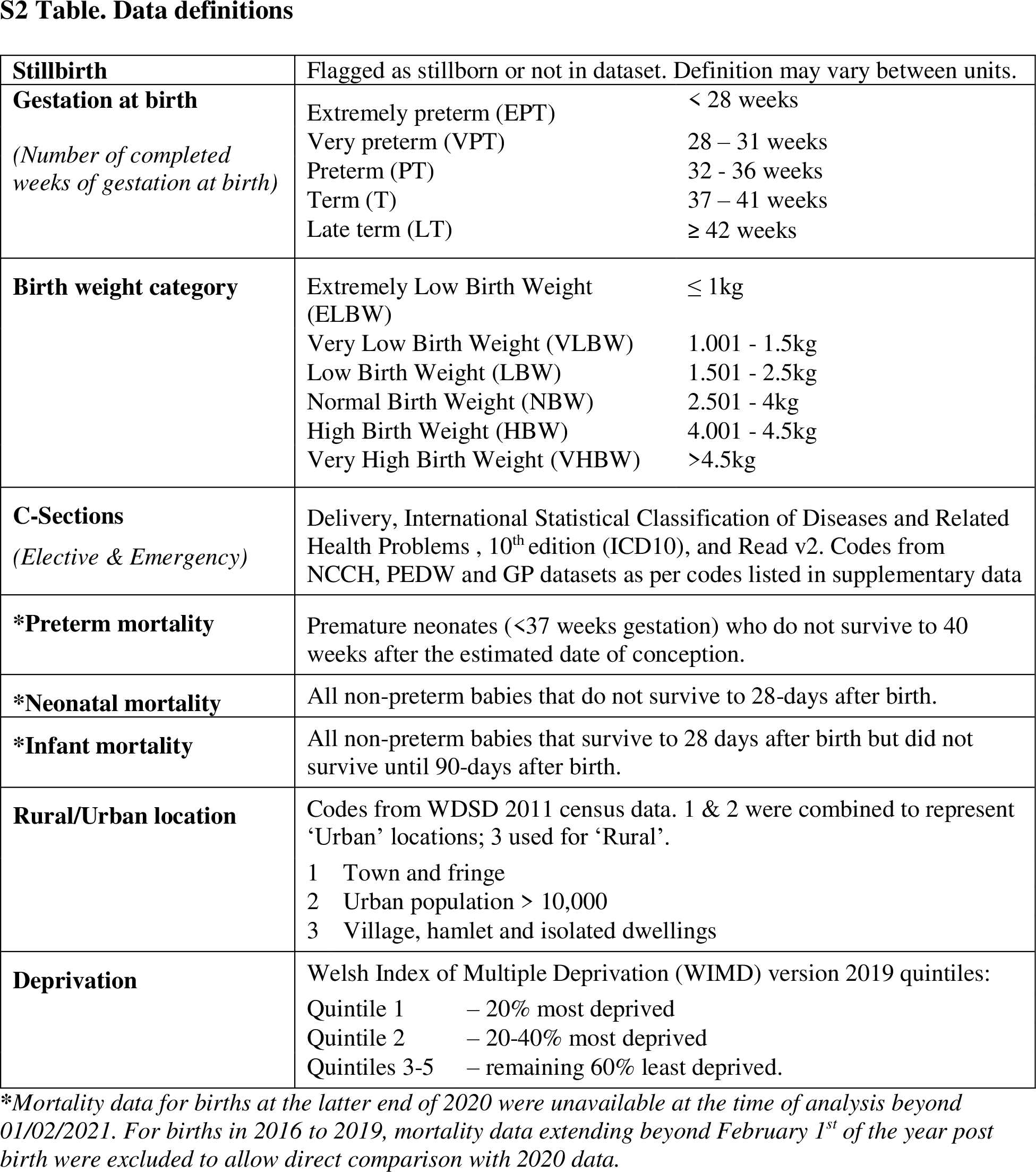

Supplement: S2 Table — (TIF) [file pone.0267176.s002.tif]

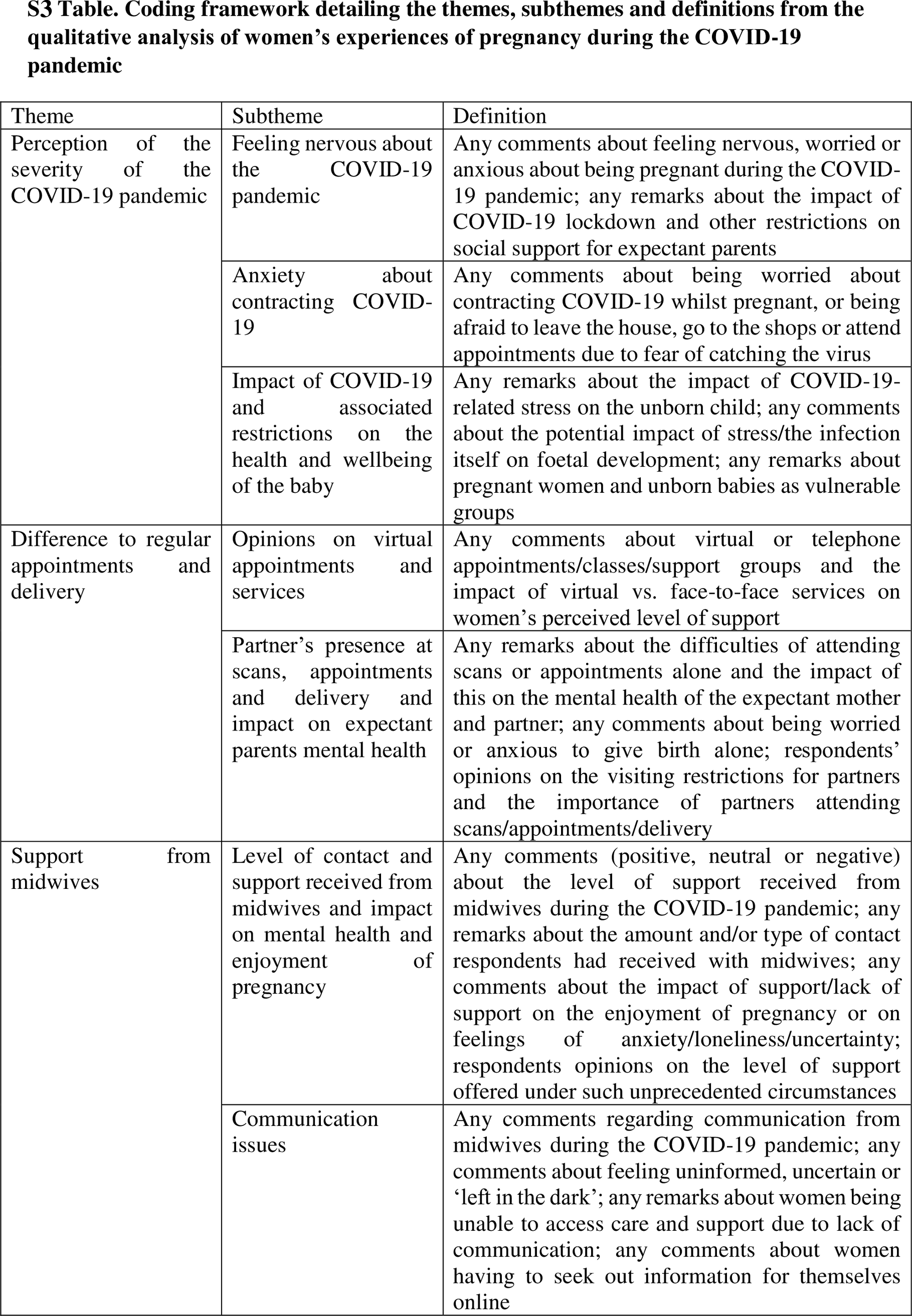

Supplement: S3 Table — (TIF) [file pone.0267176.s003.tif]

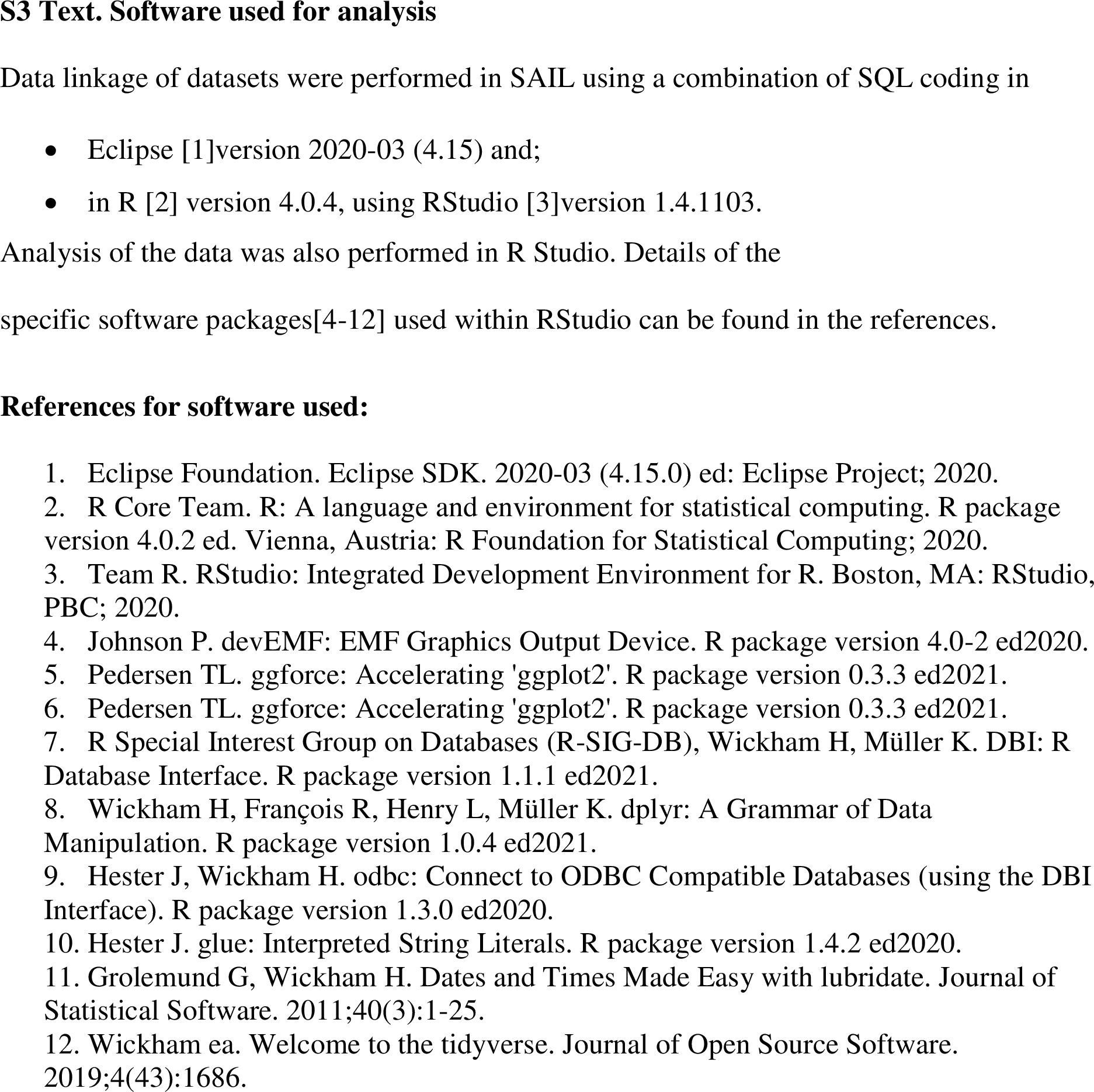

Supplement: S1 Text — (TIF) [file pone.0267176.s004.tif]
