# Supplementary material for: Population birth outcomes in 2020 and experiences of expectant mothers during the COVID-19 pandemic: A ‘born in Wales’ mixed methods study using routine data
Source: PLoS One. 2022 May 24;17(5):e0267176. doi: 10.1371/journal.pone.0267176 (PMC9129046; doi:10.1371/journal.pone.0267176)
Supplement: Attachment — Submitted filename: Response to Reviewers.docx [file pone.0267176.s005.docx]

Reviewer #1 comments

This is a mixed-methods study with quantitative evaluation of perinatal outcomes (including analysis stratified by rural/urban geography, deprivation indices, gestational age and birthweight) and childhood immunization delays during the pandemic, as well as qualitative evaluation of maternal stress and anxiety. Inclusion of evaluation of childhood immunizations and maternal stress/anxiety is novel and of great interest compared to prior studies published mostly on perinatal outcomes during the pandemic vs. pre-pandemic epochs. I was hoping there would be additional analysis performed connecting maternal/stress anxiety (could code as categorical variables) & association with maternal outcomes among the 215 respondents surveyed, but recognize the small “n” would limit statistical significance and power of this analysis compared the large sample size including in the initial quantitative analysis of nearly 30,00 women.

- **We agree that the small n would limit statistical significance in this study. But we do intend to do this in the future in terms of follow-up with a larger sample size and have included the following** **“further analysis in the first year of life and beyond will be needed to examine if stress in pregnancy has longer-term consequences for the infant and their family.”**

Abstract/Introduction

In background (both in abstract and page 5 of introduction), suggest specifying primary childhood immunisations.

- **This change has been made**

Methods

State 95% confidence intervals used with p-value used to determine significance (presumable 0.05).

- **This has been added**

Include description of post-hoc Poisson analysis in methods

- **This has been added – line 180 in tracked manuscript**

Page 8, Deprivation Quintiles: What socioeconomic factors are included with this deprivation index?

- **Added – line 175 of tracked manuscript**

Consider analysis of maternal/stress anxiety & association with adverse perinatal outcomes.

- **Please see above**

Results

Page 12: was December 2020 still a lockdown period in Wales?

- **The preceding statement referred to a second lockdown in December. This has been removed as it is not relevant in the sentence and the picture of lockdowns is more difficult. November comprised the latter part of a short lockdown along with some easing of restrictions leading into lockdown mid-December. Summarily greatly restricted throughout but not officially in lockdown throughout November-December.**

Do the authors have information on sociodemographic factors by which subgroup analyses could be performed? Recommend providing a table of baseline study characteristic including patient demographics for both the larger ~29,000 patients and 215 patients with qualitative analysis

- **Demographic data for mothers has been included in additional tables for the linked data and another for the qualitative participants. Additional detail has been added to the table for birth outcomes which details the deprivation levels, home environment and ethnicity.**
- **Ethnicity of non-white births is unlikely to be large enough for any sub-group analysis to be useful. Less than 8% are non-white and about 30% without an ethnicity.**
- **Without an a-priori sub-group analysis proposed would be uncomfortable to perform one now. Did the referee have a specific question they felt would strengthen the interpretation?**

Discussion

Consider additional discussion of results related to rural/urban analysis and deprivation indices. Do the authors propose potential hypotheses for their findings of reduced preterm birth in the higher deprivation group for November 2020?

- **Added the following to the discussion: With the frequently changing picture of pandemic restrictions throughout 2020 and the nature of this descriptive analysis, it would be impossible to form any valid conclusions about causal relationships for any of the effects seen. One can only speculate on reasons for the outcomes observed. It is possible that disruption to healthcare services could have impacted on lengthening the gestations at delivery and urban/rural could indicate the differences caused by proximity to such services e.g. delays to induction of labour. Other speculation could include restrictions to the lifestyles of expectant mothers – possible outcomes could be reduction of other background stressors e.g. work, appointments, day-to-day movements. Though initial anxiety at the very start of the pandemic could have cause additional anxiety prior to a period of adjustment to the new norm and an improved understanding/awareness of COVID-19. A reduction in November 2020 urban preterm births coincided with having just completed a ‘firebreak’ lockdown, and a period of easing restrictions up to the Christmas period.**

Table 2: specify definitions for extreme preterm, very preterm, moderate-late preterm etc. either in the methods or as a footnote; specify definitions for extreme low birth weight, low birth weight, high birth weight, etc.

- **Added a footnote to the table with the definitions.**

Re-label Tables 1, 2, 3 in the order in which they appear. I believe Table 1 with qualitative data should be Table 3 as it appears last

- **Done**

Figure 2 showing longitudinal time course of preterm births my months in line graph form is new compared to other studies and helpful presentation of temporal trend.

- **Thank you very much.**

Reviewer #2 comments

This paper covers several important topics; to investigate birth outcomes in 2020 compared to 2016-2019, experiences of expectant mothers during the COVID-19 pandemic as well as how immunisations have been provided during the pandemic compared to the previous year. However, my major concern regards the broad span of research questions that are addressed. I would suggest the authors to either focus on the birth outcomes, the qualitative part of the paper with expectant mothers’ experiences or the immunisations.

- **The qualitative part of the paper tells us how pregnant mothers are feeling about their pregnancies during a pandemic. By including it alongside the birth outcomes it should go some way to reassure mothers that, on balance, birth outcomes are no worse than pre-pandemic. It may help alleviate some worry, anxiety and stress. Although immunisations may seem unrelated – it may help to indicate differences which could relate to whether or not mothers’ experiences of pregnancy and birth have adversely affected their willingness to vaccinate their babies or if service disruption has caused delays. No firm conclusions are able to be drawn but it may provide a useful snapshot of pregnancy during the pandemic in Wales. Presenting only one of these components paints a very sparse picture**.

Introduction

Due to the fact that the paper is aiming to cover a number of research questions, I found that the introduction is somewhat straggling and incoherent. At the same time there is no mention of immunisations in the introduction. In the aim the authors mentions “vaccination rates” which the readers may interpret as COVID-19 vaccination rates. I assume that the authors here imply routine immunisations in childhood? Please clarify.

- **This has been changed to read primary childhood immunisations and vaccine information has been added into the introduction**

Page 4, para 1, row 3: How does “Maternal stress and anxiety during pregnancy is also known to be associated with adverse neonatal and obstetric outcomes [7,8,9]” differ from “The relationship between prenatal anxiety and obstetric outcomes is less clear [19,20]”. This is a bit unclear to me, and could either be shortened or explained better.

- **Wording has been changed to help clarity**

Page 4, para 2: missing a dot after reference 22.

- **Added**

Page 5, para 1: add “2020” after March for clarification.

- **Added**

Page 5, para 1: Please rephrase the sentence starting with “September to December increased…” as I don’t think the months actually increased but potentially the cases.

- **Wording changed**

Page 6, para 1: missing a dot after reference 38.

- **Added**

Methods

Study design part 1): The authors state that the data is “routinely available”. What does this mean? Can anyone use the data? Please move the section on “total population linked data” to the beginning of the methods for coherency as these results are presented before the survey online.

- **This means it is an analysis of data which is routinely collected. Researchers can apply to access and use any of the routinely collected datasets that are held in the Secure Anonymised Information Linkage (SAIL) databank. The section population linked data moved to the beginning for coherency**

The final sentences on page 7, last para, are unclear and need to be clarified and revised: “Missing data was treated as missing except in the case of missing gestational age and, therefore, gestational age category (missing in 1327 [0.8%] cases). As the majority of the birth weights in these cases were consistent with term weights, these were assigned as 40-weeks gestation and ‘term’ category respectively.”

- **Reworded so hopefully it helps clarify. Effectively a term birth or ‘normal’ birth is considered normal by omission in a lot of cases whereas a premature baby’s GA will dictate much more variation in medical management, drug treatment, feeding, etc. so these will have the GA well documented.**

Using this type of design, comparing the years 2016-2019 with 2020, how can you know that the potential difference in outcomes is related to the pandemic? What about temporal or seasonal trends in general? Would it be possible to use another approach such as difference-in-difference model as suggested by Been JV, et al. Impact of COVID-19 mitigation measures on the incidence of preterm birth: a national quasi-experimental study. The Lancet Public Health. 2020;5(11):e604-e11.

- **It may be possible to use an alternative approach but we believe our analytical approach is appropriate for the aim of this descriptive study, which was to compare population birth outcomes in 2020 with previous years. We did not aim to investigate whether any differences were likely caused by the pandemic or other causes.**

The same thing goes for childhood immunizations. How can we know that potential differences is due to COVID-19 measures and not just a coincidence or trend that was initiated before the pandemic?

- **We agree - we are not looking for causal relationships but are looking at differences pre-pandemic vs during a pandemic – we do not believe that with observational data we can prove a causal relationship.**

In addition, on page 8, para 5, it states that “all babies have received recommended immunisations”, does this mean that the coverage of routine immunisations in childhood in Wales is 100%? This would of course be amazing, but is it really correct? Is this the same with the following doses?

- **Thank you for this comment, I have amended the statement referred to and given additional, supporting information in the discussion. National reporting data from PHE and NHS Wales support that more than 95% of children under one year of age in Wales have received all of their scheduled immunisations. We recognise gaps in the data where an immunisation scheduled for 8-weeks of age may be recorded but those due at the same time were not. The 100% arises from the assumption that if any immunisation is due at that time, all other scheduled immunisations were also given but not adequately recorded. The same assumption was made for each scheduled interval of 8-, 12- and 16-weeks.**

How did you decide on 28-days for to be “on time”?

- **Prior to 2020, Public health Wales and NHS 111 Wales published their guidance for dosing at 2-, 3-, and 4-months – not weeks as is standard elsewhere in post 2020 in Wales. This would mean that a dose given in the second month after life (up to 28 days) would still be on time. Switching to 7 days based on weekly timings would change the goalposts for previous years. I have updated this explanation and included referenced to the sources in the Methodology under ‘Total population linked data’ – line 193 in the tracked changes manuscript.**

Study design part 2: Do you have any knowledge about how those responding to the online survey differed from those not participating in the online survey? For instance, which demographic characteristics were collected and could these be compared with other pregnant women? This should be deliberated in more depth in the discussion.

- **The average age of those who completed the survey was 32, and only 29 years in routinely collected data. The percentage white was 94% compared to 92% in the routine data. This suggests the survey was slightly skewed to older, white mums.**

As the manuscript covers several outcomes, a lot of results are being presented and there is a need to structure the text somewhat for the reader to follow. I also suggest to reduce the number of outcomes and restrict the paper to one method. In addition, it seems like some of the statistical analyses that are mentioned in the results section is not part of the method section? Eg. the post hoc join point Poisson regression.

- **Sub headings have been added for structure. The post hoc information has been added**

Minor:

•Make sure that the tables are described in the right order. Now table 2 and 3 comes before table 1. Either revise the order of the tables or change the numbering of the tables so that the birth outcomes are table 1 and so forth.

- **Changed**

•Only present one decimal for percentages in the tables.

- **The larger percentages have been amended to 1 decimal place at the top of table 1 but the figures for stillbirths and the confidence intervals would not easily show any differences as, although small, these have been derived from per 1000 figures and thus a 0.01% could represent up to 300 babies in a given year.**

•There is no need to present 3 decimals for non-significant p-values (eg page 7; p=0.785)

- **This has been amended**

•Page 11, para 2: Are these results possible to see somewhere? Which table is the text referring to and how does the results imply this?

- **Added the percentages difference and reference to table one. Referred to a 2.17% reduction in emergency C-sections which largely accounts for the 2.35 % increase in unknowns.**

•Page 11, para 3: a dot is missing in the p-value, and a “s” after odd(s) ratio.

- **This has been amended**

•Page 12: Should “stratified by rural/urban” be a subheading?

- **This has been added**

•There is an inconsistency in the use of small and capital letters in “Figure/figure/Table/table”.

- **This has been amended**

•Table 3: there are some missing commas (eg. 30263). Furthermore, in the text to the table on page 13 it states that uptake of immunisations was 100% in both 2019 and 2020. In the table it looks like 91.8% were vaccinated. It should be clarified which the source population is and if it is correct that all babies born in 2019 were vaccinated.

- **The missing commas have been added. The methodology has been updated to clarify how uptake was derived where recording is likely to be erroneous. The statement has been amended to clarify that this refers to whether or not the vaccines were given - not whether they were given on time. The table caption states that the figures/percentages shown relate only to those given on-time. Hence the difference between 100% received and 91.8% received on time.** •Some of the sentences are started with a percentage, eg “25%” on page 14. I would suggest to revise these sentences or write out the number in letters.
- **This has been amended**

Discussion

What do the authors believe is the reason for the results of increased numbers of late term births mean? Chance finding? Reduced number of inductions due to lack of staff? Please elaborate.

- **Detail added. It is possible that disruption to healthcare services could have impacted on lengthening the gestations at delivery and urban/rural status could indicate the differences caused by proximity to such services e.g. delays to induction of labour possibly resulting in increased late term births.**

On page 17 it is stated that “This finding is likely related to increased interventions in preterm births and thus recording them as live births rather than stillbirths [52]; rather than any effect of 2020 lockdown.”. What does this mean? Are there live births that are mistakenly recorded as stillbirths?

- **The definition of a livebirth is complex, and clinicians may or may not record a birth as a stillbirth, or a livebirth, depending on a number of factors; including the gestational age of the baby, the parents’ wishes, and by their profession. We know there are inconsistencies across the UK as to whether extremely preterm infants are reported as a miscarriage or registered as a live births and that alongside increasing interventions in these most preterm infants there appears increasing reporting of livebirths at borderline viable gestations. We have expanded the discussion with some recent references to help clarify this point.**

On page 19, first row it is stated “There was consensus early in the study with a majority reporting a negative experience of pregnancy in lockdown.” What does this imply? Please clarify.

- **Early on in recruitment to the survey, we recognised that expectant mothers were commenting on the negative aspects of being pregnant during the pandemic**

The major limitation I see is the possibility of temporal and even seasonal trends in preterm birth for instance and I suggest that authors to further investigate the possibility of seasonal trends being the reason for their findings.

- **We did not investigate temporal/seasonal trends except in the one instance where there was a known issue with how mortality is reported in births as detailed in the paper. We agree that effects could be temporal and will consider this for future analyses. We have amended the manuscript to clarify our aim to provide only a descriptive comparison between 2020 birth outcomes and before, and not to link causality to the pandemic or any other relationship.**

Minor: Page 18, first para, last row: “is” should be replaced with “in”?

- **Done**

Conclusion

The conclusion states that further analysis in the first year of life will be needed to examine if stress in pregnancy has longer-term consequences for the infant and their family. I would think that analyses of first year of life won’t be enough for longer-term consequences.

- **Agreed but the outcomes for infants e.g. infant mortality refer to the first year of life – and for most of the 2020 cohort there is only up to 3 months worth of data. This is what the statement refers to. We did not set out to look at longer-term outcomes in any part of this study but, yes, if we were to look at longer-term consequences, this would be true. This is an ongoing research project so longer term outcomes can be investigated with the routine data and we have amended the text to reflect the need for further longer-term outcomes.**
