# Supplementary material for: Population birth outcomes in 2020 and experiences of expectant mothers during the COVID-19 pandemic: A ‘born in Wales’ mixed methods study using routine data
Source: PLoS One. 2022 May 24;17(5):e0267176. doi: 10.1371/journal.pone.0267176 (PMC9129046; doi:10.1371/journal.pone.0267176)
Supplement: Attachment — Submitted filename: Response to Reviewers.docx [file pone.0267176.s006.docx]

**Response to reviewers**

**Reviewer #1 Comments:**

**• Suggest reference to “pregnant persons” instead of pregnant “women” throughout the manuscript to increase inclusivity.**

Thank you for this comment and your feedback. However, we feel that changing the terminology to “pregnant persons” may be unclear or confusing for readers who may not know if this relates to the one who is pregnant or the partner as they are also expecting. After discussions with midwives, they agree that pregnant women or expectant mum is the appropriate terminology.

**• The introduction includes hardly any reference to childhood immunizations, nor relevant tie in to the novelty proposed by the authors in the introduction (and discussion) that we need to better understand how stress among pregnant persons is associated with this change in birth outcomes during the pandemic. I would recommend removing any research/data related to immunizations from this manuscript as this topic seems it could and should be its own separate manuscript. It is too much to have three separate research questions (birth outcomes, immunizations, stress/experiences) in one manuscript, and therefore leads to a lack of focus. This recommended change by a reviewer was not adequately addressed in this revision.**

Thank you for this comment. The introduction states “there are a lack of studies examining experiences of pregnancy during the pandemic in combination with national data on objective measures of obstetric, neonatal and infant outcomes including immunisation uptake which may potentially indicate disruption to usual access to healthcare” and we feel this provides a justification for investigating immunisations in this piece of work. We believe this research would lose something by removing all information about immunisations during the pandemic. We believe the research on immunisations is important and should be in the public domain and is fundamentally linked to the experiences and outcomes for pregnant women through COVID.

**• Also, I recommend presenting the survey data regarding birthing people’s experiences (this is the exposure of interest assessed which should precede the outcomes of interest i.e. the birth outcomes), then outcome data reported second.**

This has been amended so the survey data is presented first in the methods and results

**• Can some of the introduction be moved to the conclusion? Typically introduction should be kept to 1-1.5 pages and present a succinct, clear rationale for the manuscript with relevant background. The introduction here can be tightened up to include only the most pertinent details.**

Some of the introduction has been removed to make it more succinct and clearer

**• Line 135—remove additional period after “missing”**

This has been amended

**• Line 142: It is inappropriate phrasing to compare outcomes to a year (2020). Specify that you are comparing pre-pandemic outcomes to outcomes in the 2020 pandemic epoch.**

The wording has been changed here

**• Line 267: Add bracket in front of OR.**

This has been amended
